# Pathogenicity and virulence of *Clostridium perfringens*

**DOI:** 10.1080/21505594.2021.1886777

**Published:** 2021-04-12

**Authors:** Iman Mehdizadeh Gohari, Mauricio A. Navarro, Jihong Li, Archana Shrestha, Francisco Uzal, Bruce A. McClane

**Affiliations:** a Department of Microbiology and Molecular Genetics, University of Pittsburgh School of Medicine, Pittsburgh, PA, USA; b California Animal Health and Food Safety Laboratory, School of Veterinary Medicine, University of California Davis, San Bernardino, CA, USA

**Keywords:** *Clostridium perfringens*, toxins, sporulation, quorum sensing, two-component regulatory systems, gas gangrene, enteritis/enterocolitis, enterotoxemia

## Abstract

*Clostridium perfringens* is an extremely versatile pathogen of humans and livestock, causing wound infections like gas gangrene (clostridial myonecrosis), enteritis/enterocolitis (including one of the most common human food-borne illnesses), and enterotoxemia (where toxins produced in the intestine are absorbed and damage distant organs such as the brain). The virulence of this Gram-positive, spore-forming, anaerobe is largely attributable to its copious toxin production; the diverse actions and roles in infection of these toxins are now becoming established. Most *C. perfringens* toxin genes are encoded on conjugative plasmids, including the pCW3-like and the recently discovered pCP13-like plasmid families. Production of *C. perfringens* toxins is highly regulated via processes involving two-component regulatory systems, quorum sensing and/or sporulation-related alternative sigma factors. Non-toxin factors, such as degradative enzymes like sialidases, are also now being implicated in the pathogenicity of this bacterium. These factors can promote toxin action *in vitro* and, perhaps *in vivo*, and also enhance *C. perfringens* intestinal colonization, e.g. NanI sialidase increases *C. perfringens* adherence to intestinal tissue and generates nutrients for its growth, at least *in vitro*. The possible virulence contributions of many other factors, such as adhesins, the capsule and biofilms, largely await future study.

## Introduction

The Gram-positive, spore-forming bacterium *Clostridium perfringens* is considered to be anaerobic since it cannot grow in the presence of air [1]. However, compared to most other anaerobes, this bacterium is relatively resistant to killing by oxygen [1]. *C. perfringens* is also an unusual anaerobe by possessing an extremely rapid doubling time; its short generation time of <10 min [1] contributes to virulence by allowing this bacterium to quickly reach pathogenic burdens in foods, in wounds, or in the intestine. *C. perfringens* lacks flagella but exhibits gliding motility mediated by type IV pili, which also contribute to other potentially virulence-related functions like biofilm formation and adherence [2].

**Figure 1.**
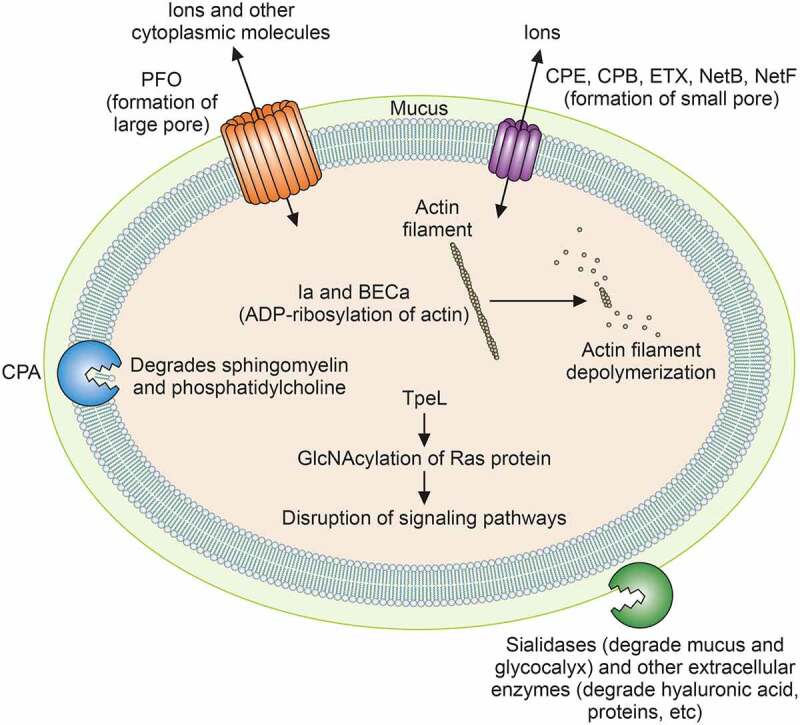
**Actions of *C. perfringens* toxins and degradative enzymes**. The cellular sites of action and mechanisms of action of major toxins and sialidases are depicted. See text for details

Although it has a ubiquitous environmental presence in decaying vegetation, soil, feces, and the normal gastrointestinal (GI) tract microbiota of humans and other animals, *C. perfringens* is also a major pathogen of humans and livestock. The virulence of this bacterium can be ascribed, in large part, to its armory of ~20 potent toxins (Table 1 and Figure 1). Toxin production patterns vary considerably among different strains, providing the basis for a recently revised [3] classification scheme (Table 2) that assigns *C. perfringens* isolates to one of the seven toxin types (A-G). The importance of toxins for *C. perfringens* pathogenicity is apparent from the association of specific toxin types with different disease niches (Table 3, with discussion later). For example, type A strains lacking any toxin-encoding plasmids cause gas gangrene (myonecrosis) and other histotoxic infections. However, those strains are not a major cause of GI disease and, in fact, are commonly present in the normal GI microbiota of humans and other animals. Except for type F strains carrying a chromosomal enterotoxin gene (*cpe), C. perfringens* strains usually need to acquire a plasmid-borne toxin gene in order to cause GI disease, as discussed later.

**Table 1.** Characteristics of toxins and extracellular degradative enzymes produced by *C.* *perfringens*

Toxin/Enzyme	Biological activity^a^	Cellular target^a^	Molecular size (kDa)^a^	Pore size (nm)^b^	Number of monomers forming the pore^b^	Gene location^c^
CPA	Phospholipase C and Sphingomyelinase	Plasma membrane	42.5	-	-	Chromosome
CPB	Pore-forming toxin	Plasma membrane	35	1.2	7?	pCW3-like plasmid
ETX	Pore-forming toxin	Plasma membrane	33	1	7	pCW3-like plasmid
ITX	Actin-specific ADP- ribosyltransferase	Cytoskeleton	Iota-a: 47.5 Iota-b: 71.5	~1	7	pCW3-like plasmid
CPE	Pore-forming toxin	Plasma membrane	35	1.4	6	Chromosome or pCW3-like plasmid
NetB	Pore-forming toxin	Plasma membrane	33	1.8	7	pCW3-like plasmid
NetF	Pore-forming toxin	Plasma membrane	31.7	4-6	6-9	pCW3-like plasmid
NetE	Putative pore-forming toxin	Plasma membrane	32.9	ND	ND	pCW3-like plasmid
NetG	Putative pore-forming toxin	Plasma membrane	31.7	ND	ND	pCW3-like plasmid
BEC	Actin-specific ADP-ribosyltransferase	Cytoskeleton	BEC-a: 47 BEC-b: 83	-	-	pCP13-like plasmid
TpeL	Ras-specific mono-glucosyltransferase	Rho-signal transduction	~206	-	-	Plasmid
CPB2	Putative pore-forming toxin	Plasma membrane	28	ND	ND	pCW3-like or pCP13-like plasmid
PFO	Pore-forming toxin; cholesterol- dependent cytolysin	Plasma membrane	54	25-45	40-50	Chromosome
Delta toxin	Pore-forming toxin	Plasma membrane	32	4-5	7?	Plasmid
NanI	Sialidase	Mucus/Surface glycolipids	77	-	-	Chromosome
NanJ	Sialidase	Mucus/Surface glycolipids/glycoproteins	129	-	-	Chromosome
NanH	Sialidase	Mucus-Surface glycolipids/glycoproteins	43	-	-	Chromosome
Kappa toxin	Collagenase	Surface glycolipids/glycoproteins	~ 80	-	-	Chromosome
Mu toxin	Hyaluronidase	Hyaluronic acid	182.6	-	-	Chromosome
Lambda toxin	Protease	?	36	-	-	pCW3-like plasmid
α-clostripain	Cysteine Protease	?	59.6	-	-	Chromosome

^a^Compiled from [6,8,22,37,45,59,64,68,72,82,255,293–296]

^b^Compiled from [5,28,31,36,58,60,68,73,78,297–301]

^c^Compiled from [12,62,109,112,115,120,123,136–144,302,303]

**Table 2.** Current *C. perfringens* toxinotyping scheme

Toxinotype	Toxin produced					
	CPA	CPB	ETX	ITX	CPE	NetB
A	+	-	-	-	-	-
B	+	+	+	-	-	-
C	+	+	-	-	±	-
D	+	-	+	-	±	-
E	+	-	-	+	±	-
F	+	-	-	-	+	-
G	+	-	-	-	-	+

**Table 3.** *C. perfringens* toxinotype: disease associations

Toxinotype	Diseases and species affected
A	Gas gangrene of humans and several animals; possible involvement in enterotoxemia and GI disease of ruminants, horses and pig; hemorrhagic gastroenteritis in dogs and horses
B	Lamb dysentery
C	Hemorrhagic and necrotizing enteritis of several neonatal animals; struck; enteritis necroticans (pig-bel, Darmbrand) in humans
D	Enterotoxemia in sheep, goats and cattle; enterocolitis in goats
E	Possible involvement in gastroenteritis of cattle and rabbits
F	Human food poisoning, antibiotic associated diarrhea and sporadic diarrhea
G	Necrotic enteritis of poultry

This review will briefly update current knowledge of *C. perfringens* pathogenicity, including the structure, action, and genetics of its toxins, which have been the traditional focus of *C. perfringens* research. Additionally, studies are now exploring the contributions of cell/tissue adherence, degradative enzymes, and sporulation to *C. perfringens* virulence, so those emerging topics will also be discussed.

## Proven or potential virulence factors

This section will briefly introduce the proven or potential virulence factors of *C. perfringens*. The role of proven, or strongly suggested, virulence factors in disease will then be discussed later in the molecular pathogenesis section.

*Major Toxins Used for* C. perfringens *Isolate Typing*

**Alpha toxin** (CPA). Virtually all *C. perfringens* isolates produce CPA, a zinc-containing phospholipase C enzyme of 370 amino acids that consists of a membrane-binding C-domain composed of a β-sheet, a catalytic N-domain composed of α-helices, and a central loop domain containing a ganglioside GM1a binding site [4]. Based on their amino acid sequences and/or antigenic cross-reactivity, CPA is related to the phospholipases of *Clostridium bifermentans, Clostridium novyi, Clostridium absonum* and *Clostridium barati* [5].

CPA is thought to use ganglioside GM1 as a receptor [4]. Through the hydrolysis of phosphatidylcholine and sphingomyelin in the plasma membrane, CPA induces the formation of diacylglycerol (DAG) and ceramide (CER), respectively [4]. Furthermore, through interactions with G_i_ type GTP-binding proteins, CPA can activate endogenous host enzymes with similar phospholipase and sphingomyelinase activities [4]. In addition to these enzymatic events in the plasma membrane, binding to GM1 allows CPA to interact with the tropomyosin receptor kinase A (TrkA), leading to activation of the MEK/ERK pathway [4]. Different intricate molecular pathways and cellular responses associated with CPA have been extensively reviewed, including cell death and the production of reactive oxygen species (ROS) or IL-8 [6].

**Beta toxin** (CPB). *C. perfringens* type B and C isolates produce CPB as a pro-toxin of 336 amino acids, containing a 27-amino acid signal sequence that is cleaved during secretion. This results in a mature toxin of ~35 kDa [7]. CPB is a β-pore-forming toxin (PFT) of the α-hemolysin family and shares amino acid sequence similarity with the β-PFTs of *Staphylococcus aureus* [8].

There is some controversy regarding CPB receptors. An early study reported that the P2X_7_ receptor is the CPB receptor for cultured THP-1 monocyte cells [9]. For example, HEK-293 cells do not naturally bind CPB but were shown to acquire the ability to bind CPB and form a CPB oligomer when transfected to produce P2X_7_. In addition, siRNA knockdown of P2X_7_ in THP-1 cells was demonstrated to reduce CPB binding and CPB oligomer formation in those naturally CPB-sensitive cells. Moreover, it was shown that CPB specifically binds to immobilized P2X_7_ receptors *in vitro* and colocalizes with the P2X_7_ receptor on the THP-1 cell surface.

More recent studies reported that platelet endothelial cell adhesion molecule-1 (CD31 or PECAM-1) is a CPB receptor on endothelial cells [10]. For example, that study reported ectopic expression of CD31 in naturally resistant mouse epithelial cells renders those cells sensitive to CPB due to their acquisition of the ability to bind CPB and form CPB oligomers. In addition, knocking out CD31 production caused mice to be insensitive to an i.p. injection of CPB. Finally, co-immunoprecipitation results using HEK 293 FT cells expressing high levels of CD31-GFP supported physical interactions between CD31 and CPB.

One possible resolution of those apparent discrepancies could be that CPB receptors vary among different cell types and/or species.

CPB is an oligomerizing toxin that forms small functional pores in the plasma membrane of susceptible cells [11]. These pores allow the entry of Ca^2+^, Na^+^ and Cl^−^ into the cells, inducing cell swelling [12]. In porcine aortic endothelial cells, CPB induces rapid disruption of the actin cytoskeleton, cell shrinkage, and cell border retraction [13]. Efflux of potassium also occurs through these CPB pores, inducing phosphorylation of p38 MAP and JNK kinases, both of which can activate pathways associated with host cell adaptation and survival [14]. Cell death induced by CPB in porcine endothelial cells exhibits features of necroptosis, since it is inhibited in the presence of necrostatin-1, a RIP1 inhibitor [15]. Nevertheless, RIP1 can also be involved in apoptosis [16].

The formation of CPB oligomers (but not CPB binding) in THP-1 cells was reduced by knockdown of Pannexin 1 production [17]. On this basis, it was suggested that CPB binding to the ATP-gated P2X_7_ receptor triggers ATP release from cells through the ATP channel Pannexin 1 [9,17]. It was also hypothesized that this ATP release could promote further CPB oligomer formation and cytotoxicity [17].

CPB is extremely sensitive to trypsin and other intestinal proteases [18]. For this reason, trypsin inhibitors are required for CPB to retain activity *in vivo* [18]. CPB variants have been identified that display different trypsin sensitivity and cytotoxic effects *in vitro* [19].

***C. perfringens enterotoxin*** (CPE). CPE, a 35 kDa single polypeptide, is produced by all type F strains and by some type C, D, and E strains. This toxin lacks primary amino acid sequence homology with other toxins but belongs structurally to the aerolysin β-PFT family [20,21]. The CPE protein consists of a C-terminal receptor-binding domain and an N-terminal cytotoxicity domain that mediates oligomerization and membrane insertion during pore formation [22].

The cellular action of CPE starts with its binding to receptors, which include certain claudins [23–25]. Claudins are a large family of proteins important for maintaining the structure and function of tight junctions made by epithelial and endothelial cells. The CPE binding ability of claudins varies considerably. For example, CPE binds to claudins-3 and −4 with high affinity, to claudins-8 and −14 with moderate affinity, and to claudins-1 and −2 poorly or not at all [25]. Claudins contain two extracellular loops (ECLs), both of which participate in CPE binding [26]. However, ECL1 is largely conserved among all claudins, so the ability of a claudin to serve as a CPE receptor largely depends upon the more variable ECL2. The presence of an Asp residue in ECL2 is important for claudins to bind CPE with moderate to high affinity [27].

Once bound to a claudin receptor, CPE becomes localized in an ~90 kDa small complex that also contains a receptor claudin and the nonreceptor claudin-1 [28,29]. Approximately six small complexes oligomerize to form a ∼450 kDa prepore on the plasma membrane surface [28]. Each CPE in the prepore then extends a β-hairpin loop that assembles into a β-barrel that inserts into the membrane to create a 1.4 nm pore [30,31]. The association of claudin-1 with the CPE pore contributes to complex stability and resistance to trypsin [29].

The CPE pore is permeable to small molecules, particularly cations [22]. Treatment with low CPE doses causes a limited Ca^2+^ influx that induces a mild calpain activation to trigger a caspase 3-mediated apoptosis [32,33], while higher CPE doses cause a large Ca^2+^ influx that induces a strong calpain activation and leads to MLKL-dependent necroptosis [33,34]. RIP1 and RIP3 are also involved in both CPE-induced apoptosis and necroptosis [34]. Dying CPE-treated Caco-2 cells also develop morphological damage, which exposes the basolateral cell surface and facilitates the formation of a ∼600 kDa CPE complex containing the tight junction protein occludin, along with receptor claudins and claudin-1 [28,29]. Formation of the ~600 kDa complex could contribute to tight junction disruption, trigger internalization of occludin and claudins, and/or increase paracellular permeability changes. It is notable that the ~450 kDa CPE complex forms both in Caco-2 cell cultures and the intestines, but the ~600 kDa CPE complex has only been detected in Caco-2 cells [35].

**Epsilon toxin** (ETX). ETX, produced only by type B and D isolates, has structural similarity to the aerolysin toxin produced by *Aeromonas* sp., so it is classified as a β-PFT of the aerolysin family [36,37]. While there is still some uncertainty about the ETX receptor, the Myelin and Lymphocyte (MAL) protein is emerging as a strong receptor candidate [38]. After binding, ETX forms a heptameric prepore [39,40], followed by the insertion of a β-barrel that allows efficient pore formation [41].

ETX is released as a weakly active prototoxin of ~33 kDa. Several intestinal proteases such as trypsin, α-chymotrypsin, and carboxypeptidases remove N-terminal and C-terminal residues from this protoxin, resulting in a mature, active protein that is 1000 times more toxic than the prototoxin [42,43]. Some *C. perfringens* strains can self-activate their ETX, an effect sometimes involving the production of sufficient amounts of λ-protease (also known as λ-toxin since it can cause edema in mice) to cleave between the 10^th^ and the 11^th^ amino acid residues from the N-terminus of the prototoxin [42,44]. Using caprine intestinal contents, it was shown that host proteases process the ETX prototoxin in a step-wise fashion, producing three ETX species with varying C-terminal residues, each of which is cytotoxic [43].

**Iota toxin** (ITX). ITX, a binary toxin produced only by *C. perfringens* type E strains, is comprised of an enzyme component (Ia) and a binding component (Ib) [45]. Removal of a ~ 20 kDa N-terminal fragment by trypsin or chymotrypsin is required to produce an active Ib, which is initially synthesized as an inactive toxin of ~100 kDa [46]. These proteases also cleave off small peptides (9 to 13 amino acid residues) from the N-terminus of the Ia precursor, producing an active form [46]. In addition, λ-protease produced by some *C. perfringens* type E strains may also activate ITX [46].

The lipolysis-stimulated lipoprotein (LSR, also known as angulin-1) is a cellular receptor for Ib [47]. This toxin component binds to the LSR N-terminal 10 to 15 residues, followed by endocytosis of ITX (together with LSR) into trafficking endosomes [48]. It has also been demonstrated that ITX entrance into host cells involves cell-surface antigen CD44-associated endocytosis [49].

Once bound to its receptor, the Ib binding component oligomerizes into heptamers that insert into the plasma membrane of target cells to form functional channels, facilitating the movement of ions and the translocation and endocytosis of the Ia enzymatic component [6,50–52]. After endocytosis, Ia translocates from late endosomes into the cytoplasm where it exerts ADP-ribosylating activity involving the covalent attachment of ADP-ribose onto an Arg at residue 177 of actin [53,54]. This effect induces depolymerization of actin filaments, which increases the presence of G-actin monomers [55,56].

By ADP-ribosylating actin, iota toxin changes cell morphology and disorganizes intercellular tight and basolateral junctions, producing an increased paracellular permeability in cultured intestinal cells *in vitro* [50]. Mechanisms of cell death associated with ITX in target cells involve features of necrosis and apoptosis [6,57].

**Necrotic enteritis B-like toxin** (NetB). NetB, a 33 kDa single-chain protein [58], is a member of the α-hemolysin family of β-PFTs. NetB shares homology with several pore-forming toxins, including 40% identity to *C. perfringens* delta toxin and 38% and 31% similarity with CPB and *S. aureus* alpha hemolysin, respectively [58,59]. Like other members of the α-hemolysin PFT family, the NetB monomer has four domains, including the β-sandwich, latch, rim, and pre-stem domains [60].

The NetB receptor has not yet been identified. NetB forms heptameric pores with an internal diameter of approximately 26 Å on susceptible cell membranes and the β-barrel channel of this pore has a strong preference for cations [60,61].

*Other Toxins Not Used for* C. perfringens *Isolate Typing*

**BEC toxin**. BEC (binary enterotoxin of *C. perfringens*) is a novel binary clostridial toxin made by type A *C. perfringens* strains associated with some human foodborne gastrointestinal disease [62,63]. Like ITX, BEC is a member of the actin-ADP ribosylating toxins family. BEC consists of two independent components, i.e. an enzymatic effector component (BECa) and a cell-binding component (BECb), which display 44% and 43% amino acid sequence identity to iota toxin Ia and iota toxin Ib, respectively [62]. The BECb receptor has not yet been identified.

It was shown that culture supernatants of BEC-positive strains cause fluid accumulation in rabbit intestinal loops [62]. In addition, it has been suggested that the BECb component is responsible for most of BEC’s enterotoxic activity since a *becB* null mutant lost fluid-accumulating activity in the suckling mouse model. However, complementation of this null mutant was not reported [62], so further research is required to understand the importance of BEC-producing *C. perfringens* in human foodborne gastroenteritis.

**Beta2 toxin** (CPB2). CPB2, a 28 kDa pore-forming toxin [64], was first identified in a *C. perfringens* isolate obtained from a piglet suffering from necrotic enteritis [64]. Subsequently, the gene (*cpb2*) encoding this toxin has been detected in isolates from a wide variety of animals and humans with enteric disease, including humans, cattle, sheep and goats, horses, chicken, and swine [65].

Most or all *C. perfringens* types can produce CPB2, which has no significant amino acid homology with CPB [64]. There are two major *cpb2* variants, the “consensus” gene or the “atypical” gene [66]. The consensus gene is almost always expressed in porcine isolates (>90%), but about half of the non-porcine isolate *cpb2* genes were shown to have a frameshift that leads to lack of expression [66]. However, a later study showed that the majority of atypical genes in isolates from a wide range of domestic animals are actually expressed [67].

The mode of action and receptor for CPB2 remain unclear at present.

The involvement of CPB2 in intestinal diseases of different animal species also remains unproven, since *cpb2*-positive *C. perfringens* strains are commonly isolated from healthy animals [65]. In the absence of any evidence to the contrary, it should be regarded as an accessory and minor toxin.

**Delta toxin**. Delta toxin is a 32 kDa single-chain protein produced by some type B and C strains. The delta toxin gene (*cpd*) appears to be located on plasmids that have not yet been characterized [68]. This toxin belongs to the α-hemolysin branch of the β-PFT family. Delta toxin displays significant homology with other members of this family, such as CPB (43% identity), NetB (39.6% identity), NetF (39% identity), and alpha-toxin of *S. aureus* (32% identity) [68,69].

Ganglioside GM2 has been suggested as a potential cell-surface receptor for delta toxin [68,70]. Delta toxin generates relatively large pores (~4 nm) on biological membranes based upon hemolysis inhibition techniques [68]. Recently, it was determined that this toxin can cause fluid accumulation and intestinal damage in mice intestinal loops [71]. However, further investigation is required to understand whether this toxin, when produced, contributes to the pathogenesis of type B- and C-associated diseases.

**NetF toxin**. NetF, a PFT made by some type A strains, is a 31.7 kDa single-chain protein that belongs to the α-hemolysin family of β-PFTs [72]. Like NetB, NetF shares some amino acid homology with other α-hemolysin family toxins including 48% identity to NetB, 39% identity to Delta toxin, 34% identity to CPB, and 30% identity to *S. aureus* alpha-toxin [72].

Recently, it was shown that NetF binds to a sialoprotein(s) on the surface of biological membranes and form pores containing 6–8 NetF monomers [73]. Osmotic protection assay results revealed that the NetF pore has a functional diameter of ~4-6 nm in RBCs and equine ovarian cell lines, which is larger than the NetB pore (1.8 nm) and *S. aureus* alpha-toxin pore (2.8 nm) [58,73,74].

**Perfringolysin O** (PFO). PFO (also referred to as theta toxin) is produced by most *C. perfringens* strains, with the exception of type F strains that have a chromosomal *cpe* gene or type C Darmbrand strains [75]. PFO is a PFT and the prototype of cholesterol-dependent cytolysins, a family that includes toxins produced by several Gram-positive bacteria [36,76]. The synthesized toxin contains a 27-amino acid signal peptide, while the mature secreted protein consists of 472 amino acids of ~53 kDa [76]. PFO has an elongated rod shape that is rich in β-sheets and it is mostly hydrophilic [36]. Four domains can be recognized in the PFO molecule, of which, the fourth domain, located in the C-terminal part, contains three loops that are involved in the binding to cholesterol present on target cells [77]. The high affinity of PFO for its cholesterol receptor is involved in concentrating the toxin in cholesterol molecules arranged in arcs on the plasma membrane, allowing oligomerization and membrane insertion [78].

The model of PFO pore formation involves the binding of water-soluble PFO monomers to cholesterol in plasma membranes, which is mediated by the L1-L3 loops from domain 4 of PFO [77]. The resultant large pores in the plasma membrane induce cell lysis by a colloid osmotic mechanism [79]. Though PFO is able to induce or interfere with intracellular signaling, including the SUMOylation pathway [80], its main activity is related to alteration of the membrane integrity [36].

**TpeL toxin**. Toxin perfringens large (TpeL), a ~ 205 kDa protein, belongs to the clostridial glucosylating toxin [81,82] family that also includes the *Clostridioides difficile* toxins A and B (TcdA/TcdB), *Clostridium sordellii* lethal and hemorrhagic toxins (TcsL and TcsH), and *Clostridium novyi* alpha toxin (Tcnα) [83,84].

The cell-surface receptor for TpeL is the low-density lipoprotein receptor-related protein 1 (LRP1) [85]. TpeL causes an N-acetylglucosaminylation of Ras proteins at threonine 35, thereby inactivating these small GTPases and inducing myriad signaling effects that lead to inflammation and cell death [82].

The timing of TpeL production is controversial, with some reports indicating TpeL is made during sporulation but others linking it to vegetative growth [86–88]. Isolation of *tpeL*-positive type G strains from chickens with necrotic enteritis has led to speculation about its accessory role in necrotic enteritis [89]. Subsequently, studies showed that *tpeL-* and *netB*-positive type G strains are more virulent than *netB*-only type G strains [90].

Whether TpeL contributes to pathogenicity, when produced, remains to be elucidated.

### Degradative Enzymes

*C. perfringens* produces a vast array of extracellular degradative enzymes, such as proteases (e.g. clostripain), hyaluronidase (mu toxin), collagenase, and endoglycosidases. Virulence contributions of these factors are now coming under intensive study. For example, it has been established that the endo-N-acetylgalactosamindase EngCP [91], but not the protease clostripain [92], is important during gas gangrene caused by type A strains, while two zinc metalloproteases contribute to avian necrotic enteritis caused by type G strains [93].

The best-studied *C. perfringens* degradative enzymes are the sialidases (neuraminidases), which generate free sialic acids (nine carbon, negatively charged terminal sugar residues) from various sialoglycoconjugates found on host cell surfaces or mucus [94,95]. *C. perfringens* produces three sialidases named NanJ, NanI and NanH. NanI (77 kDa) and NanJ (129 kDa) are secreted exosialidases, while NanH (43 kDa) is a cytoplasmic sialidase, at least during early growth [96].

All three sialidases share the same family 33 carbohydrate-binding module, while the two exosialidases contain additional accessory carbohydrate-binding modules [95]. Due to those differences in their carbohydrate-binding modules, the three sialidases possess some variations in their properties [96]. For example, compared to NanJ and NanH sialidases, NanI exhibits more heat tolerance [96]. The three sialidases also show different substrate preferences and vary in their sensitivity to various metal ions [96]. However, all three sialidases work best at low pH (pH 5) and are sensitive to p-chloromercuribenzoate, which reacts with thiol groups in proteins [96].

Most *C. perfringens* strains produce all three sialidases and, for those strains, NanI is their major sialidase [94,97,98]. The exceptions are type F strains that have a chromosomal *cpe* gene and type C Darmbrand strains, which typically do not carry the *nanI* gene [99]. However, those *nanI*-negative strains do possess the *nanH* gene, sometimes along with the *nanJ* gene [99].

### Spore Resistance

Spores facilitate the survival of *C. perfringens* in harsh environments. Of particular note are type C Darmbrand strains and type F strains with a chromosomal *cpe* gene, both of which cause human food-borne illnesses (discussion later in the *C. perfringens* disease/epidemiology section) and form especially resistant spores [100,101]. Their highly resistant spores likely contribute to foodborne pathogenesis by increasing survival of these strains against food environment stresses, such as low or high temperatures, osmotic pressure, chemical preservatives, and pH extremes [100–103].

To illustrate the exceptional heat resistance properties of their spores, type C Darmbrand strains or type F chromosomal *cpe* strains produce spores with a D_100_ (the time required at 100°C to reduce spore viability by one log) of ≥30-120 min [100,101]. In contrast, spores made by other *C. perfringens* strains have a D_100_ value of <5 min [100]. The highly resistant spores of type C Darmbrand and type F chromosomal *cpe* disease strains also show exceptional resistance to other food environment stresses, e.g. when stored at the low temperatures found in refrigerators or freezers for 3–6 months, these spores exhibit an average log reduction in viability of only 0.3 or 0.6 at, respectively, 4°C or −20°C [103]. Similarly, the highly resistant spores of some foodborne disease isolates exhibit unusually strong resistance to food preservatives like nitrite, osmotic stress, and pH extremes [102].

Major contributors to *C. perfringens* spore resistance are the α/β-type small acid-soluble proteins (SASPs). These SASPs bind to spore DNA and provide protection from various environmental stresses. *C. perfringens* produces four major SASPs, each of which contributes to spore resistance against heat, chemicals, and UV radiation [104,105].

Multilocus Sequence Typing (MLST) analyses determined that the chromosomal *cpe* type F food poisoning strains and type C Darmbrand isolates, both of which produce exceptionally resistant spores, represent a distinct genetic cluster within *C. perfringens* [101]. Those type C or F strains producing highly resistant spores also make a unique SASP4 variant that has, at residue 36, an Asp vs. the Gly consistently present at this residue in the SASP4 of *C. perfringens* isolates producing more sensitive spores [104]. Based upon results of studies using SASP4 knockout mutants complemented to express either the Asp36 or Gly36 SASP4 variant, the SASP4 Asp36 variant is very important for the exceptional spore resistance properties of chromosomal *cpe* type F strains or type C Darmbrand strains, i.e. in the same strain background, production of the Asp36 SASP4 variant significantly increases spore heat, nitrite, and cold resistance compared to the production of the Gly36 SASP4 variant [101,104,106]. The mechanism behind this resistance enhancement involves the SASP4 Asp36 variant binding more efficiently and tightly to spore DNA compared to the Glu36 SASP4 variant, an effect that offers more protection against stress-induced DNA damage [106]. Interestingly, SASP4 binds preferentially to AT-rich DNA sequences, while SASP2 binds better to GC-rich DNA sequences [106]. Since the *C. perfringens* genome contains >70% AT, it is not surprising that SASP4, particularly the SASP4 Asp36 variant, plays such an important role in spore resistance.

SASPs are not the only factor determining spore resistance properties. Factors such as spore coat thickness, spore core size, the concentration of DPA and metal ions, and the protoplast-to-sporoplast ratio have also been implicated in *C. perfringens* spore heat resistance [107,108].

### Adhesins

Several *C. perfringens* proteins, including collagen adhesion protein (CNA) and fibrinogen-binding proteins FbpA and FbpB, have been suggested to function as adhesins during disease (see the molecular pathogenesis section).

## *C. perfringens* Genetics

### Genome

In 2002, *C. perfringens* (Strain 13, a type A isolate) became the first Gram-positive anaerobic bacterium within the phylum Firmicutes whose genome was fully sequenced [109]. A recent large-scale comparative analysis of 56 closed and draft sequences of *C. perfringens* from a wide range of toxinotypes (A-G) revealed these strains contain a single circular chromosome of 2.9–4.1 Mb with relatively low G + C% content (averaging between 27.7% and 28.7%) and encoding between 2600 and 3800 predicted genes [110]. The larger genome size of the draft sequences compared to the closed chromosome relates to the carriage of large plasmids, particularly in type B-G strains. Analysis of those *C. perfringens* genomes indicated the pangenome consists of 11,667 genes (12.6% core genes and 87.4% accessory genes), suggesting considerable genomic diversity among *C. perfringens* strains, putting it on a par with the genome of *Escherichia coli* [111]. Most variable and unique regions in the accessory genome of *C. perfringens* are associated with mobile genomic elements, such as insertion sequences (ISs), transposases, and prophages [110,112–115]. Unlike ISs and transposases, there is no strong evidence for prophage-associated genes playing a crucial role in *C. perfringens* virulence. In addition, the lack of hallmark differences in GC skew signs of horizontal gene transfer (HGT) and the fairly uniform nucleotide compositions of these variable regions suggests that, i) gene acquisition in these strains is not a recent event, ii) *C. perfringens* only maintains those acquired genes with a high degree of nucleotide composition similarity to the chromosome and/or iii) these genes were acquired only from very closely related organisms with low G+C % content [112].

Analyses of the 240 publicly available complete or draft *C. perfringens* genomes, along with unassembled short read sequences, revealed the presence of multiple putative novel toxin homologs with amino acid sequence identity to well-characterized *C. perfringens* toxins such as CPE, CPB, ETX, and ITX [116]. Those observations suggest that more genetic plasticity and virulence protein diversity remains to be identified in this bacterium.

*C. perfringens* requires a number of essential nutrients and amino acids for proliferation [117]. Genome sequencing explained these requirements by showing that *C. perfringens* lacks genes required for the biosynthesis of many amino acids. It also lacks genes for the tricarboxylic acid cycle. However, the *C. perfringens* genome encodes degradative enzymes like sialidases and a complete set of enzymes for fermentation and glycolytic pathways, which facilities the utilization of complex host carbohydrates by their degradation into simple sugar components [109]. In addition, *C. perfringens* carries more than 200 transport-related genes (e.g. ABC transporters) for sugars, amino acids, nucleotides, and anions/cations. These features enable this bacterium to overcome its inability to synthesize essential carbohydrates and amino acids by acquiring these molecules from host tissues [109,110,118]. Another characteristic of the *C. perfringens* genome is the large number of encoded rRNA operons and tRNAs, which allows rapid production of secreted enzymes and toxins to support the exceptionally fast growth of the organism, an important aspect of virulence or outcompeting other bacteria involved in the decomposition of dead animals or tissue [109,119].

Although many *C. perfringens* toxin genes are carried on plasmids (see below), the chromosome does encode some proven (e.g. CPA and PFO) or potential (e.g. sialidases) virulence factors [109,112]. The availability of additional complete genomes for a variety of *C. perfringens* toxinotypes will provide a better understanding of the role of other chromosomal genes (e.g. genes encoding CNA, pili, and iron acquisition genes) in the pathogenesis of *C. perfringens*, as well as the genetic basis of host species adaptation by *C. perfringens*, where this occurs.

### Plasmids

**Figure 2.**
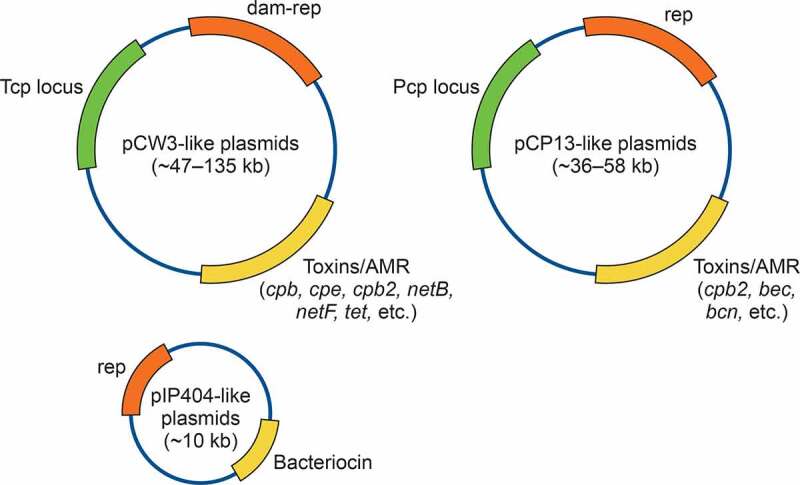
**Diagrams of the major known plasmid families of *C. perfringens***. Orange depicts the replication (rep) region, green depicts the conjugative transfer regions in pCW3-like plasmids and pCP13-like plasmids and yellow depicts the regions carrying variable genes encoding toxins, antimicrobial resistance (AMR) factors or bacteriocins. See text for further details

Plasmids play a major role in *C. perfringens* pathogenicity, particularly when this bacterium causes diseases originating in the intestines [120]. Known *C. perfringens* plasmids belong to three main families (i.e. the pCW3-like, pCP13-like, and pIP404-like plasmids, depicted in Figure 2) based upon their genes mediating the initiation of plasmid DNA replication [120–123]. Plasmids in the pCW3-like and pCP13-like families are conjugative, whereas the pIP404-like plasmid family is non-conjugative [120–123].

The pCW3-like plasmids share a common conjugation system designated as “*tcp”* (Transfer of Clostridial Plasmids) and have been identified in all seven toxinotypes of *C. perfringens* [120,124–126]. The *tcp* locus shares a low degree of sequence homology with the conjugative transposon Tn*916* that is found in a wide range of bacteria [121]. This locus encodes 11 proteins (TcpA to TcpJ and TcpM), with 9 of these proteins being required for efficient conjugation transfer of *tcp*-conjugative plasmids, including TcpA (a coupling protein), TcpC and TcpH (structural proteins), TcpD and TcpE (integral membrane proteins), TcpF (a putative ATPase), TcpG (a peptidoglycan hydrolase protein), and TcpK and TcpM (relaxosome proteins) [121,127–133].

The pCW3-like plasmids contain an ~35 to 40 kb conserved core region that encodes 22 critical genetic elements required for efficient plasmid replication, maintenance, stability, partitioning, regulation, and transfer (i.e. the *tcp* locus). Apart from this core region, each pCW3 family member also has a diverse accessory region [115,120,121,124,126,134] that can contain genes encoding virulence factors such as toxins, bacteriocins, or antibiotic resistance determinants (e.g. resistance against tetracycline or chloramphenicol), as well as other proteins with hypothetical functions.

Almost all clinically important *C. perfringens* toxin genes [115,120,124–126,135–143], can be located on *tcp*-conjugative plasmids. To date, the *cpb, etx, iap/ibp, tpeL, netF* and *netB* genes have been localized exclusively to pCW3-like plasmids (Table 4). The *cpb2* gene encoding CPB2 is also sometimes present on these plasmids, although it can also be carried by pCP13-like plasmids (described below). The pCW3-like toxin plasmid family ranges in size from ~50 to ~140 kb in size. Several of these toxin plasmids have been completely sequenced [115,137,140,142,144].

**Table 4.** Size and diversity *C. perfringens* plasmids encoding key-toxins^1.^

^1^Modified from [125]

Shared colors other than black indicate a similar/identical plasmid

*indicates sequenced plasmid from pCW3-like family

**indicates sequenced plasmid from pCP13-like family

“Seq” indicates a sequenced plasmid; numbers are size in kb

“ND” Not determined

^a^Compiled from [101,120,139,143,304]

^b^Compiled from [120,138,139,142]

^c^Compiled from [120,141,144]

^d^Compiled from [101,120,140,143,144,302,305]

^e^Compiled from [120,139,143]

^f^Compiled from [109,113,115,120,124,142,305]

^g^Compiled from [124,137,306]

^h^Compiled from [115,307]

^i^Compiled from [62]

The gene (*cpe*) encoding CPE is unusual in that it can be located on either the chromosome or plasmids [145,146]. Approximately 70% of type F human food poisoning isolates carry their *cpe* gene on the chromosome [1], where it is closely associated with flanking IS*1470* sequences that may represent the chromosomal integration of a *cpe*-carrying transposon [147]. As shown in Table 4, the remaining 30% of type F food poisoning strains, virtually all type F non-food-borne human GI disease strains and *cpe*-positive type C, D, and E strains carry their *cpe* gene on large pCW3-like conjugative plasmids. In type F strains, *cpe* plasmids mainly cluster into two sub-families [140], i.e. the pCPF4969-like plasmids (which, among other differences, do not carry the *cpb2* gene) and the pCPF5603-like plasmids (which do carry the *cpb2* gene).

Interestingly, the variable and unique regions on pCW3-like toxin plasmids are often associated with mobile genomic elements, such as ISs and transposases [120,125,126], suggesting a role for those ISs/transposases in the continued evolution of pCW3 family plasmids, e.g. these genetic elements may help to explain the variability among pCW3-like plasmids and how a single pCW3-family toxin plasmid sometimes accumulates multiple toxin genes [120,125,126]. For instance, the presence of transposase genes at both ends of the *netF* or *cpe* loci on large *tcp*-conjugative plasmids suggests that the toxin genes located in these loci are derived from a mobile element [115,140]. Interestingly, in type E strains both the iota toxin genes (*iap/ibp*) and complete or disrupted *cpe* genes are located near one another on pCW3-like plasmids [144,148]; since ISs flank those toxin genes, the adjacent presence of *cpe* and *iap/ibp* genes suggests that a possible hot-spot for IS insertion exists on pCW3-like plasmids.

An interesting feature of pCW3-like plasmids is that multiple (up to five) related but independent plasmids of this family can stably co-exist within a single *C. perfringens* cell [115,124,137,140,141,143,149]. Bioinformatic and functional studies showed that sequence differences in their partitioning and segregation genes are responsible for this plasmid compatibility [124,134]. Recent studies revealed that *C. perfringens tcp*-conjugative plasmids may have up to 10 different types of partitioning system (ParMRC_A-J_) located in the core region of these plasmids [150,151] and those variations in partitioning machinery enable a single *C. perfringens* strain to carry multiple discrete plasmids. Another intriguing feature of pCW3-like plasmids is that their replication protein is highly similar and specific to *C. perfringens* plasmids [120,121,126], which may explain why these plasmids have not adapted to other bacterial species.

Since pCW3 has a copy number of ~5 [126], the toxin plasmids of the pCW3-like family are also likely to be present at a low copy number. Consistent with that belief, there are no consistent differences in CPE production levels between type F strains carrying a single copy of the *cpe* gene on their chromosome vs. those carrying *cpe* on pCW3-like plasmids [152].

A second distinct *C. perfringens* conjugative plasmid family are the pCP13-like plasmids [123], which can carry toxin genes such as *cpb2* and *bec* (encoding binary clostridial enterotoxin). The pCP13-like plasmids are conjugative plasmids that contain a newly described conjugative locus (~27 kb) designated the “pCP13 *Clostridium perfringens”* (*pcp*) transfer locus [123]. The *pcp* locus encodes almost all of the key components required for transformation homologues of a Gram-positive type four secretion system (T4SS). Unlike the *tcp* locus, which is only found in *C. perfringens*, the *pcp*-conjugative locus appears to be evolutionarily related to the conserved conjugation system existing in other pathogenic clostridial species, such as *Clostridium botulinum, Clostridioides difficile*, and *Clostridium sordellii* [123].

The third *C. perfringens* plasmid family includes the pIP404-like plasmids. These plasmids are not conjugative. Compared to the pCW3-like and pCP13-like plasmids, the pIP404-like plasmids are relatively small and usually harbor a bacteriocin-encoding gene (*bcn*), but no toxin genes [122].

## Regulation of *C. perfringens* virulence gene expression

### Two-component regulatory systems

To control their virulence factor production, many pathogens use two-component regulatory systems (TCRS) that consist of a membrane sensor and a cytoplasmic transcriptional response regulator. Consistent with this theme, the *C. perfringens* genome encodes >20 TCRS [109,112] and two of these TCRSs have, thus far, been implicated in virulence.

The VirS/VirR TCRS is the best characterized *C. perfringens* TCRS. It is encoded by an operon and consists of a VirS membrane sensor histidine kinase and a VirR transcriptional regulator [153–155]. By regulating CPB production, VirS/VirR contributes to type C strain virulence during intestinal pathogenicity and enterotoxemic lethality [156]. Furthermore, by controlling PFO and CPA production by type A strains [157,158], or NetB production by type G strains [159], this TCRS is also likely to be important during, respectively, gas gangrene or avian necrotic enteritis. VirS/VirR is also a global regulator that controls the production of many housekeeping genes [160].

Computer modeling predicts that the VirS protein consists of seven predicted transmembrane domains, several exposed extracellular regions, and a C-terminal tail [161,162]. The C-terminal tail is located in the cytoplasm and contains several conserved motifs typical of histidine kinases, including the likely site of autophosphorylation, i.e. H255 and the G box, which is involved in ATP binding [161]. A recent study [162] implicated the 2^nd^ extracellular loop of VirS in signal sensing (see quorum sensing section). When this signal is received, VirS autophosphorylates and then activates VirR by transferring a phosphate onto a conserved aspartate residue located in the N-terminal region of VirR [161,162]. The C-terminal domain of activated VirR recognizes and binds to VirR boxes, which are two imperfect, directly repeated sequences located upstream of the target gene [163,164]. This binding directly increases the expression of some genes, including toxin genes encoding PFO and NetB [153,159,164]. Other VirS/VirR-regulated genes, such as the *cpa* gene encoding CPA, lack VirR boxes in their promoters but are indirectly controlled by small regulatory RNA molecules such as VR-RNA, whose promoter does contain VirR boxes [154].

A second TCRS named RevS/R has also recently been implicated in regulating *C. perfringens* virulence. This TCRS controls the expression of several virulence-associated genes, including several degradative enzymes such as clostripain and sialidases [165]. Consistent with that role, RevR is an important regulator of *C. perfringens* virulence in the mouse myonecrosis model [166].

### Quorum sensing (QS) systems

*C. perfringens* also uses density-sensing QS systems to regulate virulence factor production. The Agr-like system is the most important QS system of this bacterium for virulence. This QS system was identified when it was shown to control CPA and PFO production [167,174]. Consistent with those findings, the Agr-like QS system is important for *C. perfringens* to cause gas gangrene [169]. Subsequent studies established that this QS system also regulates the production of several toxins with proven involvement in *C. perfringens* intestinal diseases [168,170,171]. Studies with *agrB* null mutants [168,171] showed that this QS system is necessary for type C strains to cause necrotic enteritis and enterotoxemia in rabbits or mice, respectively, or for type G strains to cause necrotic enteritis in chickens. The Agr-like QS system is also necessary for biofilm formation by *C. perfringens* [172], which could be important during infections, although that is not yet proven.

An Agr-like QS system is found in several Gram-positive pathogens and uses an autoinducing peptide (AIP) to signal a classical TCRS [173]. In *C. perfringens*, the *agr* operon contains four genes [167,174], including one encoding AgrD, which is the precursor for the signaling peptide, and another encoding AgrB, which is likely to be an integral membrane endopeptidase (functions of the two upstream genes in this operon are unknown). After the production of the AgrD precursor peptide in the cytoplasm, AgrB is thought to process this peptide to an active AIP and then export that peptide extracellularly. In *C. perfringens*, the native AIP appears to be a 5-amino-acid peptide in a thiolactone ring [175,176].

**Figure 3.**
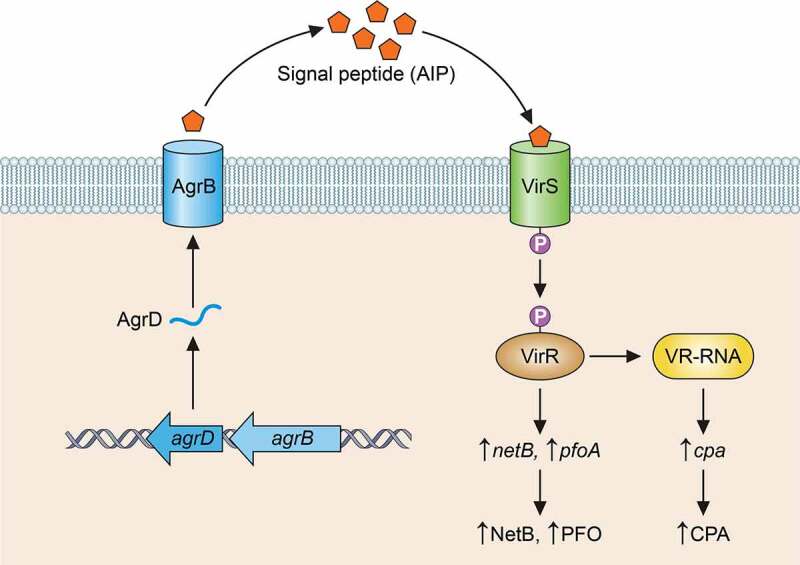
**Current model for cross-talk between the VirS/VirR two-component regulatory system and the Agr-like quorum sensing system in *C. perfringens***. The AgrD peptide is processed by AgrB (and perhaps other unidentified factors) to form a cyclic autoinducing signaling peptide (AIP). AIP then binds to VirS, which in turn phosphorylates (p) VirR. The phosphorylated VirR protein then binds to VirR boxes upstream of some toxins genes (e.g. the *pfoA* gene) and upstream of the *vrr* gene encoding VR-RNA. VR-RNA then leads to increased transcription of genes encoding toxins such as CPA. This reults in increased production of those toxins. Based upon [162,308]

The *agr* operon of the classical *S. aureus* Agr system also encodes the AgrA/AgrC TCRS, where AgrC is the membrane histidine kinase that binds the active AIP and AgrA is the response regulator phosphorylated by activated AgrC [173]. However, the *C. perfringens agr* operon does not encode an AgrA/AgrC homolog. Since the production of several *C. perfringens* toxins, including PFO, CPA, CPB, and NetB, is co-regulated by both the Agr-like QS and VirS/VirR TCRS, it was hypothesized that the VirS membrane sensor protein is an AIP receptor for the *C. perfringens* Agr-like QS system [177]; this proposal was recently confirmed when the 2^nd^ extracellular loop of VirS was shown to be involved in AIP binding [162]. A model for the interactions between the Agr-like QS and VirS/VirR TCRS is depicted in Figure 3. It has also been shown that some AIP-like mimic peptides with thiolactone rings can interfere with *C. perfringens* toxin production [169,175] and inhibit the development of gas gangrene, suggesting potential therapeutic applications.

*C. perfringens* also possesses a LuxS/AI-2 QS system that may contribute to regulating the production of CPA and PFO [178]. However, this QS system is not important for regulating CPB production or biofilm formation [168,172].

### Other regulators of toxin production during vegetative growth

Besides TCRS and QS systems, *C. perfringens* uses several other transcriptional regulators to control toxin production. CodY is a global transcriptional regulator that senses nutrient availability by binding GTP or branched-chain amino acids [179]. CodY binds to the promoter region of CodY-regulated genes when cytoplasmic levels of GTP or BCAA are sufficient, i.e. under nutrient-rich conditions. In *C. perfringens* type D strain CN3718, CodY binds to the promoter region of the *etx* gene and positively regulates ETX production [179]. In that strain, CodY did not affect PFO or CPA production levels.

CcpA (catabolite control protein A), a member of the LacI/GalR family, functions as another *C. perfringens* global regulatory protein, acting as a repressor of CcpA-regulated genes in the presence of increasing glucose levels [180,181]. It also positively controls ETX production [180] and both sporulation and CPE production [181], as described below. Interestingly, NanI indirectly production affects CodY and CcpA, which directly upregulate ETX production [180].

### Regulation of sialidase production

Production of *C. perfringens* sialidases is controlled by a complex regulatory network that includes the VirR/VirS TCRS, Agr-like QS system, ReeS, and CodY [160,165,166,179,182]. Regulator of Extracellular Enzymes Sensor (ReeS) is an orphan histidine kinase with a conserved sensor histidine kinase domain but no potential DNA binding motif [165]. Production of NanI and NanJ are both positively regulated by Rees, although a *reeS* null mutation does not significantly affect virulence in the mouse myonecrosis model [165].

However, the major regulator of sialidase production identified to date is NanR, which is present in the *nan* operon that encodes the complete pathway for transporting and metabolizing sialic acid [182,183]. In the absence of sialic acid, NanR represses *nanI* expression [183].

### Regulation of sporulation

As mentioned earlier, sporulation plays an important role in *C. perfringens* pathogenicity by enabling survival in harsh conditions like cooking of foods. Spores can also be important for the transmission of diseases like gas gangrene and type F food poisoning [184]. Sporulation is also important for the pathogenesis of type F strains since CPE synthesis is sporulation-dependent [184].

*C. perfringens* sporulation begins when one or more sporulation-specific orphan histidine kinase senses still unidentified signals and phosphorylates Spo0A, which is a transcriptional regulator essential for initiating *C. perfringens* sporulation, i.e. an *spo0A* knockout mutant is unable to form spores [185]. An orphan kinase, named CPR0195, was recently shown to be important for initiating sporulation and CPE production by type F strain SM101 in sporulation media [186]. That study also showed CPR0195 can directly phosphorylate Spo0A. Once Spo0A is phosphorylated, this leads to the production of a cascade of sporulation-associated sigma factors (σF, σE, σK and σG) that regulate the sporulation process [187,188]. Sporulation requires the production of all four sporulation-associated alternative sigma factors [187,188]. However, CPE production is independent of SigG, which is expressed in late sporulation [188]. Western blot results suggest that SigF controls the production of the other three sporulation-associated sigma factors [188]. Production of SigE and SigK directs RNA polymerase to transcribe the *cpe* gene from upstream SigE- or SigK-dependent promoters [187].

*C. perfringens* sporulation is a complex process subject to both positive and negative regulations. Besides the direct sporulation pathway regulators described above, the transcriptional regulators CcpA and CodY impact *C. perfringens* sporulation and CPE production [179,181,189], although the mechanisms involved are not yet clearly defined. Interestingly, CodY is required for the sporulation of type F food poisoning strain SM101 but represses sporulation of type D strain CN3718 [179,189]. These strain variations are due to differences in *abrB* gene expression patterns between *codY*-null mutants of SM101 and CN3718 [189], which supports the involvement of AbrB (a sporulation repressor) and/or SigH in regulating *C. perfringens* sporulation through a mechanism requiring further study. The Agr-like quorum-sensing system also participates in controlling sporulation since inactivation of the *agrB* gene in non-foodborne human GI disease type F strain F5603 reduced sporulation ~15,000-fold due, at least in part, to significant decreases in *sigF* and *sigG* expression [170]. Another sporulation regulator is a small RNA encoded by the *virX* gene. This regulatory RNA significantly inhibits sporulation and CPE production by type F food poisoning strain SM101 [190]. While *virX* expression is directly controlled by VirR/S TCRS [190], there is no direct evidence yet that this TCRS controls sporulation. Finally, the sialidase regulator NanR also positively regulates sporulation and CPE production through an unknown pathway [183].

### Regulation of germination

Germination of spores also plays an important role in *C. perfringens* pathogenesis [191]. For example, spores implanted into wounds must germinate back to vegetative cells to multiply, produce CPA and PFO, and cause gas gangrene. Similarly, food poisoning often occurs when spores in foods germinate back to vegetative cells that multiply in a contaminated food before ingestion.

Germination begins with the *C. perfringens* spore sensing small molecules named germinants that are strain-specific and include such factors as amino acids, KCl, and phosphate [191]. Those germinants bind to receptors, of which GerKC is the most important for food poisoning strains [191]. This results in CspB-mediated proteolytic activation of the cortex hydrolase SleC, which induces the removal of the cortex layer of the spore to allow hydration of the core and the resumption of metabolism [191]. Germination also involves the removal of calcium dipicolinate from the core and removal of SASPs from the chromosome [191].

## *C. perfringens* diseases/epidemiology

### Histotoxic infections

Clostridial myonecrosis or gas gangrene is a highly lethal, necrotizing infection of skeletal muscle and subcutaneous tissue that is most commonly caused by *C. perfringens*s type A [75]. The current incidence of gas gangrene in humans is low, but lethality remains relatively high [192]. With prompt diagnosis and appropriate treatment including surgical care, antibiotic treatment, and hyperbaric oxygen therapy, the lethality varies between 5% and 30% [193]. If untreated, the disease reaches 100% lethality [194]. Amputation of the affected tissue is sometimes required as a life-saving procedure [195]. Clostridial myonecrosis was a common war injury infection with an incidence of ~5%, but with improvement in disease detection and care, the incidence has fallen considerably since the Vietnam war era [193]. In the United States, the incidence of gas gangrene is about 1,000 cases per year but is higher during natural disasters such as earthquakes [192,196].

**Figure 4.**
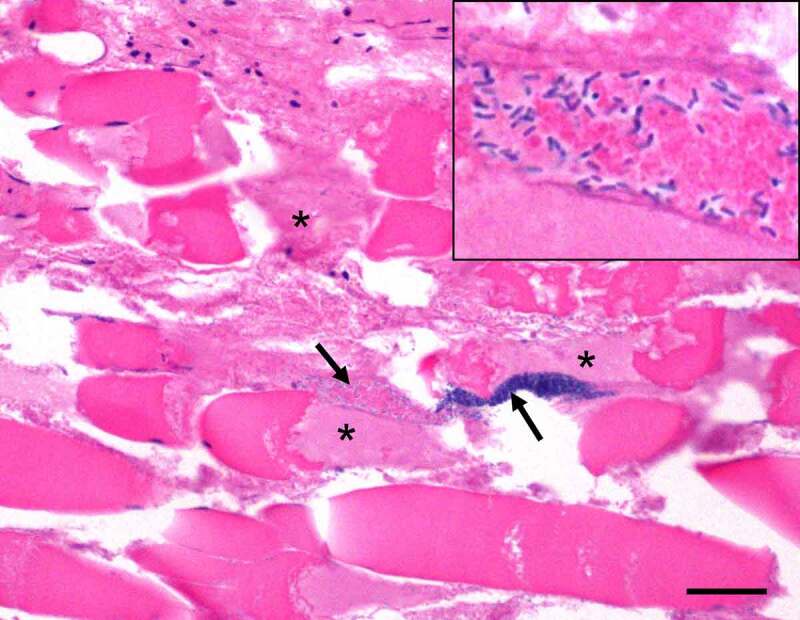
**Gas gangrene by *C. perfringens* type A in an experimentally infected mouse**. This animal was challenged with 10^9^ washed vegetative cells of *C. perfringens* strain 13 and euthanized 4 h later. There is severe skeletal muscle necrosis (asterisks) and myriad intralesional bacilli (arrows and insert). Notice that there is minimal inflammatory infiltrate. Hematoxylin and eosin. Scale bar = 50 μm

Gas gangrene involves invasion of traumatic wounds by type A vegetative cells or spores, followed by vegetative cell multiplication and toxin production (see next section), which then induce rapid, severe, and extensive necrosis in the affected tissue (Figure 4). The disease is clinically characterized by pain, local edema and emphysema, fever, and myonecrosis, which commonly progress to rapid bacterial spread leading to sepsis, toxemia, shock, and death [197]. Gas gangrene in animals may be produced by a variety of clostridia, including *C. perfringens* type A. The latter is the main cause of gas gangrene in horses [198].

### Diseases originating in the intestines

**Infections by type A strains**. Although several gastro-enteric syndromes of animals have reportedly been associated with type A strains [199], their role in these diseases is controversial. The main difficulty for ascribing disease to type A isolates is that they are ubiquitous in the environment and the intestine of many animal species. Therefore, isolation of this bacterium from GI samples has no diagnostic significance [199].

Possible exceptions are the type A isolates encoding the recently discovered NetF toxin. Those strains are suggested to be associated with canine hemorrhagic gastroenteritis and necrotizing enterocolitis in foals [72]. The link is mostly epidemiological and based on these strains seeming to be in higher prevalence in animals with those diseases [72,200,201].

**Infections by type B strains**. Type B infection is classically a disease of sheep, although rare cases have been reported in cattle and horses [202]. In sheep, the disease is known as lamb dysentery and it is characterized by necro-hemorrhagic enteritis and, very rarely, focal symmetrical necrosis that are thought to be produced by CPB and ETX, respectively [202–205]. Lamb dysentery is an example of a *C. perfringens* infection causing both intestinal lesions and enterotoxemia, where toxins produced in the intestine not only act on the intestines but are also absorbed into the circulation and then affect distant organs like the brain.

Recently, *C. perfringens* type B was isolated from the feces of a human patient with multiple sclerosis (MS) [206] and ETX serum antibodies were found in patients with this disease [207]. These findings prompted speculation that ETX may be associated with the pathogenesis of MS [206,207], although conclusive evidence is not yet available in this regard.

**Figure 5.**
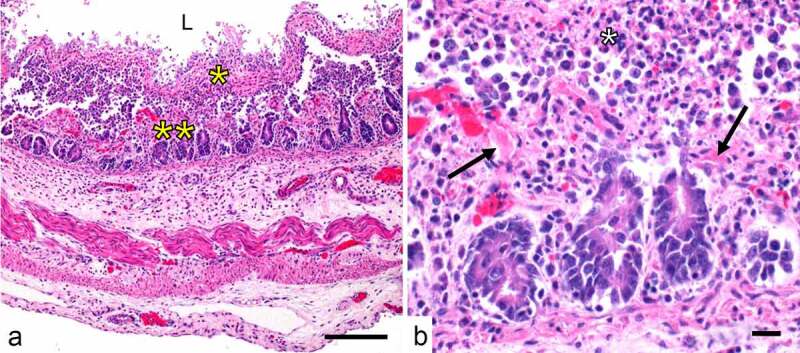
**Naturally-acquired necrotic enteritis caused by *C. perfringens* type C in a neonatal piglet**. A. Diffuse necrosis of mucosa (**), which is covered by a pseudomembrane (*) that is composed mostly by fibrin, cell debris and inflammatory cells. These effects are a consequence of CPB (see text). The intestinal lumen is indicated (L). B. This higher magnification of image A shows thrombosis of mucosal vessels (↗) and myriad neutrophils admixed with fibrin and cell debris forming a pseudomembrane (*) on the surface of the necrotic mucosa (**). Scale bar=50 μm. Hematoxylin and eosin

**Infections by type C strains**. Type C strains cause necrotizing enteritis (Figure 5) and enterotoxemia in many mammalian species including humans, with a special predisposition for neonates [208,209]. Type C strains cause necrotizing enteritis (Figure 5) and enterotoxemia in many mammalian species. In animals, they have a special predisposition for neonates, which is believed to be related to CPB sensitivity to trypsin, which is a natural defense against the disease. Because the colostrum is a potent trypsin inhibitor, neonate animals that ingest colostrum are more susceptible to the action of CPB [208,209]. A rare form of type C disease, known as “struck,” results in the sudden death of adult sheep, but predisposing factors are not understood [210].

In humans, foodborne type C disease was common in malnourished people in post-World War II Germany, where it was known as Darmbrand [101]. This disease, known as enteritis necroticans or PigBel, also had a high prevalence in the 1960s in Papua New Guinea [211–213]. Although no longer endemic, sporadic cases still occur in that country [101]. PigBel was observed to occur mostly in malnourished children with presumed low levels of trypsin due to poor diet and consumption of large amounts of sweet potatoes, which contain a powerful trypsin inhibitor [211,212]. These children then developed the disease when they ingested incompletely cooked meat (often pork) contaminated with type C strains [211,212]. Rare cases of type C infections have also been reported for people with diabetes or other pancreatic diseases [214,215].

In humans and other animals, type C disease is acute or pre-acute and highly lethal [210,214,215]. Clinically, this disease involves diarrhea and abdominal pain. Occasionally, neurologic alterations and sudden death can be seen in animals [210]. Gross and microscopic lesions are characterized by severe necrotizing enteritis or enterocolitis that starts at the villus tip in the small intestine or in the superficial mucosal epithelium in the colon. Mucosal and submucosal thrombosis are an inconsistent finding [210,216]. Lesions outside the alimentary system during enterotoxemia are nonspecific and consist of circulatory disturbances, including serosal congestion and hemorrhage, and pulmonary congestion and edema [210].

**Figure 6.**
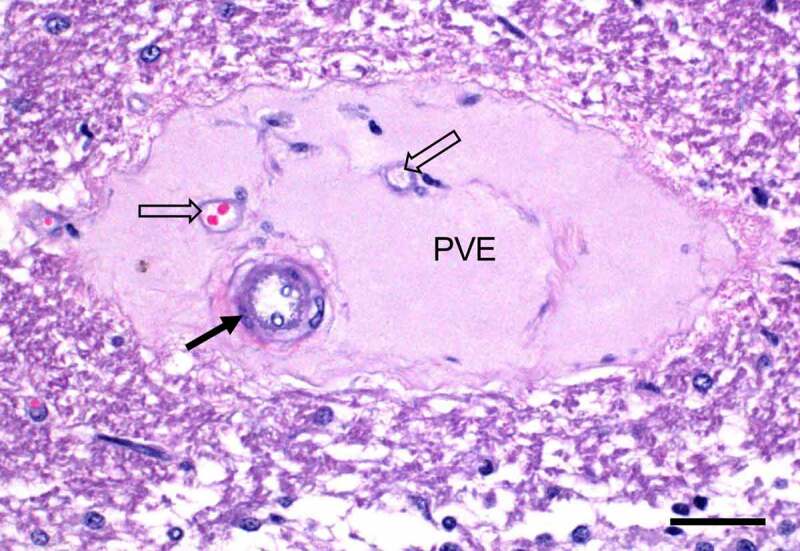
**Perivascular proteinaceous edema (PVE) in the cerebellar white matter of a sheep experimentally infected with *C. perfringens* type D**. This lesion is a consequence of the action of epsilon toxin on the vascular endothelial cells, which increases vascular permeability allowing albumin and water to leave the vascular lumen. An arteriole (solid arrow) and two venules (hollow arrows) are indicated. Scale bar = 50 μm. Hematoxylin and eosin

**Infections by type D strains**. Enterotoxemia of sheep, goats, and rarely cattle [205,217] is caused by *C. perfringens* type D. Disease caused by type D strains in sheep and cattle is a true enterotoxemia with lesions in the brain (Figure 6) and other extra-intestinal organs but only infrequent intestinal lesions. However, infection of goats with these strains causes enterocolitis with or without enterotoxemia.

In sheep, and less frequently in goats and cattle, sudden ingestion of feeds rich in highly fermentable carbohydrates [205,217] is the main predisposing factor for the disease. *In vitro, C. perfringens* type D produces more ETX in media with a low glucose vs high glucose content [218]. This effect mimics what is presumed to happen when undigested complex carbohydrates bypass the fore-stomachs and stomach of sheep in spontaneous cases of type D enterotoxemia.

**Infections by type E strains**. The role of type E isolates in human and animal disease has not been fully elucidated. Although a few cases of *C. perfringens* type E-associated disease have been described in several animal species [219,220], the great majority of those diagnoses were based on isolation of this microorganism from the intestinal content of sick animals. Because *C. perfringens* type E can be found as a normal inhabitant in the intestine of healthy individuals of many animal species, isolation of this bacterium does not fulfill the diagnostic criteria for type E disease [221].

**Infections by type F strains**. Type F strains are major gastrointestinal pathogens of humans. They cause *C. perfringens* type F food poisoning, the second most common bacterial food-borne illness in the USA, where it affects 1 million people/year and causes annual economic losses of ~$400 million [1]. The high incidence of this food poisoning is attributable to two factors. First, most type F food poisoning strains produce highly resistant spores (see the spore resistance section), which facilitates their survival in improperly held or undercooked foods, particularly the large meats (roasts or turkeys) that are often vehicles for *C. perfringens* type F food poisoning outbreaks. Second, when spores in incompletely cooked foods germinate into vegetative cells, the short doubling time of *C. perfringens* vegetative cells allows rapid attainment of a sufficient bacterial burden (>10^6^ to 10^7^ vegetative cells/gram of food) to initiate GI disease [1]. Reported outbreaks of this food poisoning are typically large, likely because smaller outbreaks often go undiagnosed [1]. Identified outbreaks often occur in institutions, which need to prepare large amounts of food in advance and then hold those foods for extended periods before serving [1].

*C. perfringens* type F food poisoning starts with the ingestion of food containing large numbers of type F vegetative cells [1]. After a brief *in vivo* multiplication, those bacteria sporulate and produce CPE in the intestines. At the completion of this *in vivo* sporulation, the mother cell lyses, which releases CPE (as well as the mature spore) into the intestinal lumen. The released CPE then binds to the intestines and exerts its action (as described later).

This food poisoning typically involves diarrhea and abdominal cramps that develop within 12–24 hours and then self-resolve within a day [1]. However, fatalities do occur in the elderly or debilitated people. In addition, several *C. perfringens* type F food poisoning outbreaks in psychiatric facilities have led to fatalities in relatively young and physically healthy people [222–224]. Those fatalities occurred in people with preexisting constipation or fecal impaction side-effects of psychoactive drugs taken for preexisting mental illness. Therefore, when these individuals acquired type F food poisoning they did not develop CPE-induced diarrhea, which normally flushes CPE from the intestines and attenuates disease. Consequently, the prolonged contact between CPE and the intestines in these people likely facilitated CPE absorption into the circulation, where it could then bind to organs like the liver and kidneys, causing a fatal enterotoxemia.

*C. perfringens* type F isolates also cause ~5-15% of all non-foodborne human GI disease cases, which include antibiotic-associated diarrhea (AAD) and sporadic diarrhea [225]. CPE-associated AAD cases are typical of longer duration (up to several weeks) and more severity than cases of *C. perfringens* type F food poisoning. CPE-associated AAD often occurs in the nosocomial environment and develops after the intestines of patients (particularly the elderly) receiving antibiotics become colonized by type F strains in the nosocomial environment and those strains then produce CPE *in vivo*. In contrast to type F food poisoning isolates, which often carry a chromosomal *cpe* gene and produce highly resistant spores, nearly all type F strains causing non-foodborne GI illnesses carry a plasmid-borne *cpe* gene and make relatively sensitive spores [145].

**Infections by type G strains**. Type G strains are responsible for necrotic enteritis (NE), one of the most prevalent diseases of poultry worldwide. Worldwide economic losses due to NE are estimated to be ~ (US) 5 USD billion [226].

Under natural conditions, infection by *Eimeria* spp. is the most common predisposing factor for NE [227,228]. Type G strains cause disease in several avian species, but no cases have been described in non-avian species. Type G NE occurs mainly in chickens, but cases have been described in many other avian species, including, amongst others, turkeys, ostriches, quail, capercaillies, geese, bluebirds, lorikeets, and crows [229].

NE usually occurs in the form of outbreaks in 2–6 week-old broiler chickens [230], a fact that has been associated with low anti-clostridial immunity during that age window, when maternal antibodies wane and before the immune system matures [231,232]. Individual cases and outbreaks have, however, occasionally been reported in chickens of various ages [229].

The disease may be sub-clinical, affecting mostly weight gain, or clinical. In the latter, clinical signs are varied and include one or more of the following: reluctance to move, diarrhea, decreased appetite or anorexia, huddling, and dehydration [229]. Occasionally, birds may be found dead without clinical signs having being observed [229].

In cases of acute NE, gross lesions are mainly seen in the jejunum and ileum, although the duodenum and ceca may also be affected. These effects consist of gas distention of the intestine, which is full of dark brown, semi-liquid content with fibrin strands, with an ulcerated mucosa covered by a fibrino-necrotizing membrane [229]. Rarely blood can be seen, although hemorrhage is not a common feature of NE. In subacute and chronic cases of NE, the lesions are similar but the intestinal wall is usually thickened. In subclinical NE, multifocal mucosal ulcerations are observed [229]. Cholangiohepatitis may be also seen grossly in the livers of some chickens with NE and is characterized by enlarged, firm, and pale livers with multiple scattered yellow necrotic foci [229,233].

**Figure 7.**
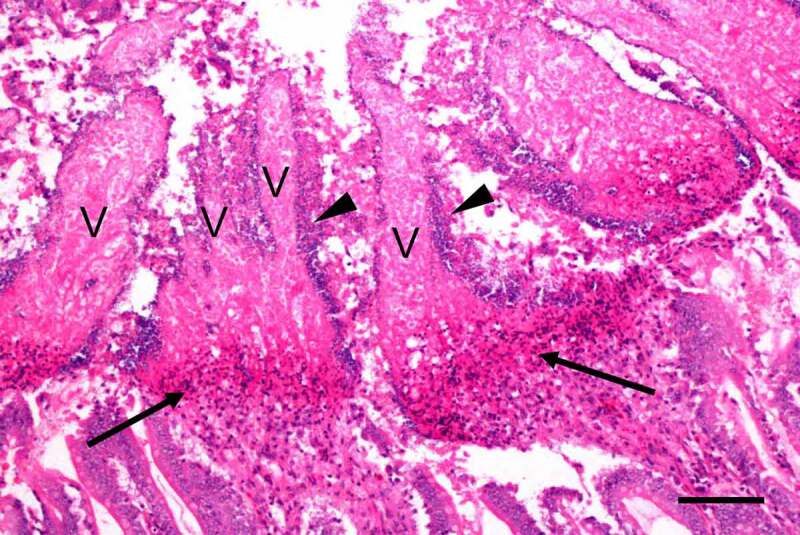
**Small intestine of a chicken with naturally acquired necrotic enteritis produced by *C. perfringens* type G**. Notice the necrotic and denudated villi (v), lined by large number of bacilli (arrow heads). A wide band of heterophils (arrows) separates the superficial necrotic villi from the more normal looking deep part of the tissue (bottom of the photograph). Scale bar = 100 μm. Hematoxylin and eosin

Microscopically (Figure 7), birds with NE have multifocal to diffuse mucosal necrosis, which, in some cases, may be transmural. When the lesions are on the surface of the intestinal mucosa, a sharp line of demarcation between necrotic and viable tissue can be seen [229]. Multifocal coagulative necrosis of the liver is frequently observed [233]. Large numbers of intralesional Gram-positive rods, which are positive for *C. perfringens* immunohistochemistry, are observed in both intestinal and hepatic lesions [229,233].

## Molecular pathogenesis of *C. perfringens* infections

Improved technology to introduce targeted null mutations in *C. perfringens* genes [234] has revolutionized our understanding of which virulence factors contribute to infections caused by the various types of this bacterium. Coupling information obtained using those mutants with results of cell biology studies using purified proteins, *C. perfringens* molecular pathogenesis is now being elucidated, particularly regarding virulence contributions of host colonization and the local or distant effects of its various toxins during disease, as discussed below.

### Colonization

The ability to colonize, i.e. persist and proliferate/survive *in vivo*, is an important feature of some *C. perfringens* infections, e.g. type F strain nonfoodborne human GI diseases. *C. perfringens* colonization typically involves nutrient acquisition for growth/survival and adherence. For example, type F nonfoodborne GI disease can persist for up to several weeks even in the face of diarrhea, indicating that intestinal adherence and nutrient acquisition must be necessary for these type F strains to stably colonize the intestines in the presence of diarrhea. However, for acute diseases such as type F food poisoning, nonadherent *C. perfringens* cells present in the lumen likely mediate disease. For type C disease, the picture is more complicated. Walker et al. reported [235] that adherent type C strains mostly adhere to necrotic lesions, which would raise the unresolved question of whether these bacteria only adhere to already toxin-damaged tissue or whether these bacteria only bind to certain intestinal regions and then toxins cause damage to that local area.

To colonize the GI tract, *C. perfringens* uses several molecular strategies, including the production of sialidases. While C. perfringens produces three sialidases (see virulence section), NanI is produced by the type F nonfoodborne GI disease strains that persist in the intestinal tract but not by most of the type F food poisoning strains that are associated with acute infections. That correlation suggests an important contribution of NanI to *C. perfringens* chronic intestinal disease [94,236]. This possibility is supported by studies using NanI mutants that demonstrated NanI contributions to type F nonfoodborne GI disease strain intestinal colonization and persistence in a mouse model [236].

When NanI-producing *C. perfringens* strains cause diseases originating in the intestines, this sialidase is present in the intestinal lumen, where it encounters host proteases such as trypsin and chymotrypsin. Interestingly, NanI is proteolytically activated by trypsin, chymotrypsin, and mouse intestinal fluids [237]. Amino acid sequencing demonstrated that this activation involves N-terminal processing of the NanI protein [237]. Those observations suggest that protease activation of NanI may further contribute to colonization caused by NanI-producing *C. perfringens* strains.

Besides their effects on increasing *C. perfringens* intestinal adherence, NanI contributions to intestinal colonization likely also involve increasing nutrient acquisition. Several *in vitro* studies have supported NanI contributions to the growth and survival of *C. perfringens* strains associated with intestinal infections. For example, NanI was shown to support the *in vitro* growth and survival of type F nonfoodborne disease strain F4969 in the presence of host mucin or cultured Caco-2 cells [238], with this growth promotion involving generation and utilization of NanI-generated sialic acid, which can then be metabolized by *C. perfringens* [182,189]. Several potential mechanisms may be involved: 1) releasing sialic acid from mucin or sialic acid-modified macromolecules from host cells, 2) exposing underlying carbohydrates and amino acids, allowing other glucoside hydrolases or proteases to hydrolyze and release nutrients for utilization, and 3) allowing the action of other enzymes to produce carbohydrates and amino acids [95,236,238].

*In vitro* studies suggest that NanI also contributes to *C. perfringens* intestinal adhesion. Compared to wild-type *C. perfringens* type D strain CN3718, an isogenic triple mutant that does not produce any sialidases exhibits significantly reduced ability to adhere to cultured human enterocyte-like Caco-2 cells [97]. Restoring production of NanI (but not NanH or NanJ) by complementation of that triple mutant yielded a significant improvement in adherence [97]. It is possible that NanI facilitates *C. perfringens* adherence by modifying the surface of intestinal cells, allowing, 1) the exposure of an unknown receptor used for binding and/or 2) the reduction of negative charges of sialic acids on this surface [97,239,240].

Besides promoting colonization, NanI may also impact intestinal infections by affecting toxin activity and production. This sialidase increases the ETX sensitivity of MDCK cells, the CPB sensitivity of HUVEC cells, and the CPE sensitivity of Caco-2 cells, with those effects due to NanI causing an increase in toxin binding levels [97,237]. This enhancement may be attributable to NanI increasing the exposure of toxin receptors on the host cell surface and/or NanI modifying the host surface to reduce charge repulsion effects. Production of NanI also upregulates ETX production by a route involving both CodY and CcpA regulators [180].

Despite its role in *C. perfringens* intestinal colonization, the involvement of NanI (or other sialidases) in the context of histotoxic infections by this bacterium is less clear. In a murine myonecrosis model, a *nanJ* and *nanI* double mutant of *C. perfringens* strain 13 remained virulent, suggesting that sialic acid metabolism is not necessary for bacterial growth or persistence in skeletal muscle [98]. Nevertheless, since the mentioned myonecrosis model involves challenge with large numbers of inoculated *C. perfringens*, contributions of NanI (such as generating nutrients) during early infection may have been masked [98].

Other factors such as adhesins are also emerging as potential contributors to *C. perfringens* colonization. For example, it has been proposed that CNA contributes to enteritis in pigs by promoting adhesion to damaged intestinal tissue, based upon the prevalence of the *cna* gene in strains isolated from porcine cases [241]. Moreover, compared to less virulent strains, *C. perfringens* strains associated with severe intestinal disease in chickens are more capable of binding to collagen types II, IV, and V [242,243]. Recent studies have also implicated fibronectin (Fn) as a possible extracellular matrix glycoprotein used by *C. perfringens* for binding, and two Fn-binding proteins have been identified on this bacterium, i.e. FbpA and FbpB [244]. In the presence of Fn, *C. perfringens* is able to firmly bind to collagen, especially types II and III [245,246]. As with many other pathogenic bacteria, that encode Fbps [247], *C. perfringens* may take advantage of Fn to facilitate host cell contact and further colonization.

### Local toxin effects

#### Gas gangrene

**Type A strains**. During gas gangrene, CPA plays an essential role [248,249] due to several cellular and tissue effects, including hemolysis [250,251], myonecrosis [248], leukostasis [252,253], platelet aggregation [254], vasoconstriction [255], and inhibition of neutrophil differentiation [256]. CPA also induces firm adhesion of neutrophils to extracellular matrix proteins and their accumulation on the vascular endothelium, explaining in part the leukostasis observed in gas gangrene [257].

PFO is also involved synergistically with CPA in myonecrosis [249] by contributing to tissue destruction and preventing bacterial lysis by host immune cells [36]. PFO is cytotoxic for polymorphonuclear leukocytes and macrophages at high concentrations, and it impairs respiratory burst, superoxide anion production, and phagocytosis of complement opsonized particles at lower concentrations [253,258]. In addition, PFO contributes to the lysis of the endosome membrane in macrophages allowing the escape of *C. perfringens* from phagosomes [259,260], and it prevents actin filament polymerization in leukocytes and migration of neutrophils [253,258].

**Type B infections**. The role of toxins in the pathogenesis of type B infections is complex and not fully understood, the general dogma being that intestinal lesions are produced by CPB, while central nervous system lesions, when present, are mediated by ETX [261]. Because ETX requires protease-activation, while CPB is inactivated by proteases, it has been postulated that in different clinical settings, either ETX or CPB (but not both) are responsible for a particular set of signs and lesions [261].

**Type C infections**. The enteropathogenicity of type C strains is mediated by CPB, a highly necrotizing toxin, as demonstrated by fulfilling molecular Koch’s postulates in rabbit small intestinal loop models [209,262]. Synergism between CPB and CPE also exists during intestinal infections by CPE-positive type C strains isolated from cases of human necrotic enteritis. This effect was shown in a rabbit intestinal loop model, and suggests that both toxins may act synergistically in some cases of human necrotic enteritis [263].

It is currently unclear which cell type in the intestines is affected first by CPB. Some evidence suggests that, early during infection, CPB affects the enterocytes of the small and large intestine [209]. For example, one study showed that intestinal epithelial damage develops rapidly (within an hour) in CPB-treated rabbit small intestinal loops, arguing that epithelial cells are a primary CPB target [264]. However, other results suggest that, in piglets, vascular endothelial cells are the primary initial target of this toxin [10]. It is possible that both epithelial and endothelial cells are targets for CPB, i.e. enterocytes might be affected first by CPB and death of these cells then allow access of CPB to the vasculature. If CPB affects endothelial cells first, the mechanism by which the toxin crosses the intestinal epithelium needs to be determined.

**Type D infections**. Molecular Koch’s postulate analyses have shown that ETX is the main virulence factor for *C. perfringens* type D strains to produce clostridial enterotoxemia in sheep, goats, and sometimes in cattle [265–267]. After *C. botulinum* and *C. tetani* toxins, activated ETX is the third most lethal clostridial toxin [65].

The action of ETX at the site of its production, the intestine, has not been well characterized. However, ETX is involved in the development of fibrinonecrotic enterocolitis in cases of enterotoxemia in goats [205], but not in sheep or cattle.

**Type E infections**. Molecular Koch’s postulates for *C. perfringens* type E and ITX have not been fulfilled in any animal species, and diagnostic criteria for the disease have not been defined.

**Type F infections**. Studies with *cpe* null mutants confirm that CPE production is essential for the GI pathogenicity of type F strains associated with food poisoning or nonfoodborne GI diseases [268].

**Figure 8.**
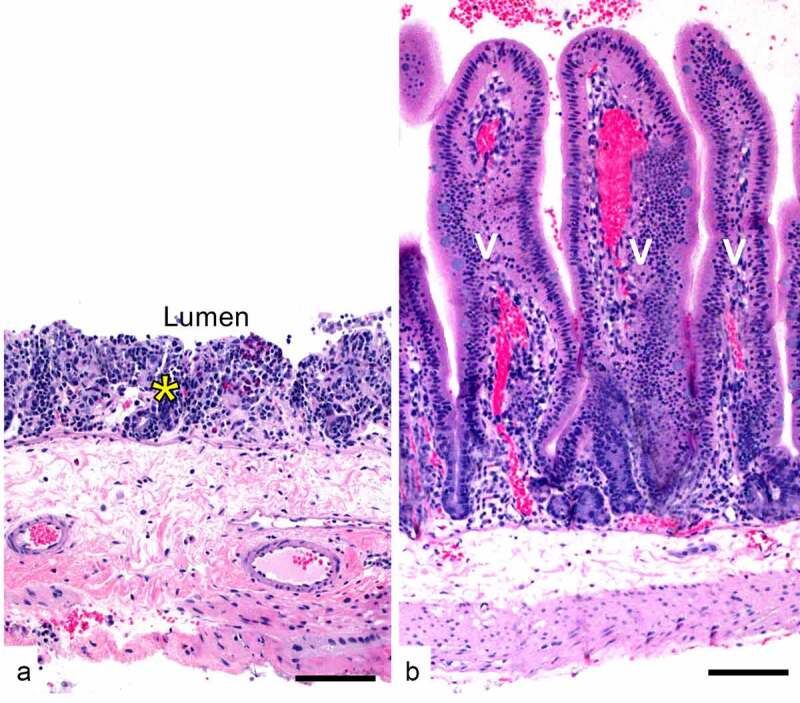
**Effect of *C. perfringens* type F enterotoxin (CPE) on the small intestinal loops of a rabbit**. A. CPE (50 micrograms) was injected into the lumen of a ligated small intestinal loop of an anesthetized rabbit. The animal was kept under anesthesia during 6 h after which it was euthanized and the intestinal loop was collected and processed for histology. There is almost complete loss of the mucosa (asterisk); no villi are observed. Scale bar = 100 μm. B. Normal control shown for comparison. This ligated small intestinal loop of the same rabbit was inoculated with buffer instead of CPE. Notice the intact mucosa with long villi (v). Scale bar = 70 μm. Hematoxylin and eosin

CPE affects the small intestine, particularly the ileum, of all tested mammalian species [269]. As shown in Figure 8, CPE-treated rabbit small intestinal loops exhibit villus shortening and epithelial desquamation [270]. This damage appears to be necessary for CPE-induced fluid and electrolyte loss into the lumen (diarrhea) since the start of this damage closely corresponds to the onset of fluid and electrolyte losses [271]. In addition, only those CPE doses capable of causing this damage could induce intestinal fluid and electrolyte losses in rabbit small intestinal loops [269]. Interestingly, CPE binds primarily to the tips of villi, yet causes destruction of the entire villus [270]. This effect may involve a bystander killing effect, whereby CPE-treated sensitive cells release a factor, possibly a < 30 kDa serine protease, to kill insensitive cells [272]. In addition to the small intestine, CPE also affects the rabbit colon *in vivo* [273] and human colonic tissue *ex vivo* [274].

**Type G infections**. Historically CPA was considered the main virulence factor when type G strains cause avian NE, but it is now clear that CPA is not essential for this disease [275]. Instead, NetB is responsible for the pathogenesis of most cases of NE [58], as established using reverse genetics, where a *netB* null mutant failed to produce lesions typical of NE in experimental chickens [58] and NetB complemented strains re-gained the capacity for this microorganism to produce NE.

Although it is currently accepted that NetB is the main virulence factor of NE-associated *C. perfringens* strains, some uncertainty remains because not all NE isolates have been found to carry the *netB* gene and a few *C. perfringens* strains isolated from clinically normal chickens also have this gene [276–279]. In addition, cases of necrotic enteritis have been described in birds from which *C. perfringens* type A, but not G, strains were isolated. Furthermore, at least one study reported that a *netB*-negative *C. perfringens* isolate reproduced necrotic enteritis when inoculated into chickens [228]. Based on these findings, while it is generally accepted that NetB has a key function in most cases of NE, other virulence factors may also be responsible for necrotic enteritis in some cases [280].

### Distant toxin effects

During gas gangrene, potent toxins (particularly CPA) eventually reach the circulation. At that time, they can cause a toxemia involving organ failure, circulatory collapse, and death [281].

Several other *C. perfringens* infections involve toxin production in the intestine, followed by absorption of that toxin to cause enterotoxemias that involve organs such as the brain, lungs, and so on. The foremost example of an enterotoxemia-inducing *C. perfringens* toxin is ETX. After production and activation in the intestine, ETX opens tight junctions [282], which may enhance its absorption into the circulation [283]. It then binds to endothelial cells in the brain, lungs, heart, and possibly other organs [284], where the main effect is to increase vascular permeability, which in turn leads to edema. In animals that survive for longer periods of time, parenchymal necrosis (mostly in the brain) develops as a consequence of the edema [285]. ETX also affects neurons and oligodendrocytes after crossing the blood-brain barrier [286–288].

Overwhelming evidence indicates that, once produced in the intestine, CPB (and perhaps other toxins made by type C strains) are also absorbed into the circulation and then act on distant organs such as the brain and lungs. For example, neurologic and respiratory signs were observed in mice challenged intragastrically with type C strains, even though those mice showed little intestinal histologic damage [18]. Furthermore, an isogenic *cpb* null mutant of a type C strain lost the ability to cause those effects, implying a major role for CPB in this type C enterotoxemia [18]. In further support of that contention, CPB can cause lethality when injected into small intestinal loops of mice even though it causes limited intestinal histologic damage [18]. Furthermore, CPB is lethal when injected intravenously [289].

Serosal hemorrhages, pulmonary edema, and hydropericardium have also been described in a natural host, i.e. goats, experimentally infected with a type C strain [290]. That pathology involves CPB since an isogenic *cpb* null mutant failed to cause the development of these signs in this model. Similar lesions are frequently observed in foals and calves naturally diseased with type C strains [208,210,216]. CPB inhibition of platelet function may be responsible for systemic hemorrhages [291].

CPE is another *C. perfringens* toxin thought to induce enterotoxemia. As mentioned earlier, type F infections in psychiatric patients can become fatal; this lethality has been attributed to the absence of diarrhea due to constipation side-effects of drugs taken by these people for their preexisting psychiatric illnesses. This absence of diarrhea is thought to prolong contact between CPE and the intestine, leading to CPE absorption into the circulation, where it then affects nonintestinal organs. Studies using a mouse intestinal CPE challenge model confirmed that intestinal CPE can be absorbed into the blood and induce enterotoxemia and that this effect causes a hyperpotassemia, which likely induces death by cardiac arrest [35].

## Summary, perspectives, and future questions

*C. perfringens* is a multi-talented pathogen, causing histotoxic infections, enteritis/enterocolitis, and enterotoxemias. This virulence versatility is attributable, in large part, to its ability to produce a panoply of potent toxins. Histotoxic infections are usually caused by relatively “simple” type A strains producing only CPA and PFO; this association probably reflects, at least in part, type A strains being the most common *C. perfringens* strains in the environment so they have the most opportunity to contaminate wounds. But is this the only explanation for this association?

To cause intestinal disease, *C. perfringens* typically acquires additional toxin genes. Many of these toxin genes are associated with ISs and present on conjugative plasmids, which provides considerable virulence plasticity when causing disease originating in the intestines. However, there may be a limitation to using this toxin plasmid strategy for intestinal virulence, i.e. due to plasmid incompatibility issues, only certain combinations of toxin genes can be maintained in a single *C. perfringens* strain. *C. perfringens* partially compensates for this drawback by using ISs to accumulate multiple toxin genes on a single plasmid. Eventually, such toxin gene accumulation on a single plasmid could give rise to novel hypervirulent strains of *C. perfringens* that do not currently exist.

Why does *C. perfringens* even produce so many toxins, particularly so many different PFTs? One insight is provided by these PFTs recognizing different receptors, which possibly allows different strains to specifically target different cell types and/or organs, particularly during enterotoxemias. Another reason for producing so many PFTs was already mentioned for type B strains, i.e. these strains produce CPB, which is sensitive to trypsin, and ETX, which is activated by GI proteases including trypsin. Thus, by producing both CPB and ETX, a single type B strain can affect a range of hosts from neonatal animals taking colostrum to adult animals with normal trypsin activity. With that said, it appears there is some evolutionary disadvantage to producing both CPB and ETX since type B strains are rare. This may be related to the necessity to maintain both CPB and ETX plasmids in a single cell, since no single plasmid has yet been identified that carries both the *cpb* and *etx* genes.

Interestingly, there can be considerable variation even among strains belonging to the same type and producing the same toxins. For example, type F chromosomal *cpe* strains and type C Darmbrand strains appear to be evolving away from other *C. perfringens* strains, including type F strains with a plasmid *cpe* gene or most other type C strains. Not only do type C Darmbrand strains and type F chromosomal *cpe* strains cluster apart from other *C. perfringens* by MLST analyses of their housekeeping gene sequences, but they also produce much more resistant spores due to their variant SASP4 and they lack the genes encoding both NanI and PFO. These strains have clearly become adapted for foodborne transmission in humans and have acquired the ability to cause acute GI infection in this host.

Many important questions remain unresolved regarding *C. perfringens* toxins themselves. For example, how does CPE damage the entire small intestinal villus even though it binds only to villi tips? Why is ETX so exceptionally potent? Why does ETX commonly cause intestinal pathology in goats but not other species? What is the connection between ETX and MS? What are the specific target cells of CPE, ETX and CPB during enterotoxemia? How do toxins like CPB and CPE cross the small intestinal epithelium? Do BEC, NetF, TpeL, CPD, and CPB2 contribute to pathogenicity, when produced? When produced by a single strain, is there any synergism between NetF and CPE in causing intestinal pathology *in vivo*? Last, close contact of *C. perfringens* with host cells upregulates the production of many *C. perfringens* toxins involved in enteritis or enterotoxemia [292], but what is the triggering host cell signal and how many *C. perfringens* regulatory pathways are involved in this upregulation?

Besides toxins, other *C. perfringens* virulence factors are also now coming under study. It has become apparent that, beyond toxins, factors (such as NanI sialidase) contribute to intestinal colonization while other factors (such as EngCP) contribute to gas gangrene. However, these studies are still in their infancy so there are also many questions remaining about non-toxin virulence factors of *C. perfringens*: Are sialidases important for intestinal virulence? Do they contribute to early steps in gas gangrene? Which adhesins are important for *C. perfringens* attachment to host tissues, particularly in the mammalian intestines? Do capsules or biofilm formation help *C. perfringens* evade immune responses? How does the presence of mucus impact *C. perfringens* growth, toxin activity and attachment to host cells? What triggers *C. perfringens* type F strain sporulation and CPE production in the intestines?

These questions will require further study to fully understand the pathogenicity of this intriguing and important pathogen.
